# Loss of immune homeostasis in patients with idiopathic pulmonary arterial hypertension

**DOI:** 10.1136/thoraxjnl-2020-215460

**Published:** 2021-05-07

**Authors:** Peter Heukels, Odilia B J Corneth, Denise van Uden, Jennifer A C van Hulst, Leon M van den Toorn, Annemien E van den Bosch, Marlies S Wijsenbeek, Karin A Boomars, Mirjam Kool, Rudi W Hendriks

**Affiliations:** 1 Department of Pulmonary Medicine, Erasmus Universiteit Rotterdam, Rotterdam, The Netherlands; 2 Department of Pulmonary Medicine, Amphia Hospital, Breda, The Netherlands; 3 Department of Cardiology, Erasmus Universiteit Rotterdam, Rotterdam, The Netherlands

**Keywords:** primary pulmonary hypertension, innate immunity

## Abstract

**Introduction:**

Autoreactivity against pulmonary vascular structures is thought to be involved in idiopathic pulmonary arterial hypertension (IPAH), but the underlying mechanisms remain poorly understood. We hypothesised that aberrant B-cell activation contributes to IPAH aetiology.

**Methods:**

Mice with enhanced B-cell activation due to B-cell-specific overexpression of the B-cell receptor (BCR) signalling molecule Bruton’s tyrosine kinase (BTK) were subjected to lung injury and examined for several pulmonary hypertension (PH) indices. Peripheral blood lymphocytes from patients with IPAH (n=13), connective tissue disease-associated PAH (CTD-PAH, n=9), congenital heart disease PAH (n=7), interstitial lung disease associated PH (n=17) and healthy controls (n=19) were characterised by 14-colour flow cytometry.

**Results:**

Following pulmonary injury, BTK-overexpressing mice showed prolonged activation of B cells and CXCR5^+^ follicular T-helper (Tfh) cells, as well as features of PH development. Patients with CTD-PAH and CHD-PAH displayed reduced proportions of circulating non-switched-memory B cells (p=0.03, p=0.02, respectively). Interestingly, we observed increased BTK protein expression in naive (p=0.007) and memory B-cell subsets of patients with IPAH and CTD-PAH. BTK was particularly high in patients with IPAH with circulating autoantibodies (p=0.045). IPAH patients had low frequencies of circulating CXCR5^+^ Tfh cells (p=0.005). Hereby, the increased BTK protein expression in B cells was associated with high proportions of Tfh17 (p=0.018) and Tfh17.1 (p=0.007) cells within the circulating Tfh population.

**Conclusions:**

Our study shows that pulmonary injury in combination with enhanced B-cell activation is sufficient to induce PH symptoms in mice. In parallel, immune homeostasis in patients with IPAH is compromised, as evidenced by increased BCR signalling and cTfh17 polarisation, indicating that adaptive immune activation contributes to IPAH disease induction or progression.

**key messages**

**What is the key question?**

- Which mechanisms are involved in the loss of immune homeostasis in idiopathic pulmonary arterial hypertension (IPAH) development?

**What is the bottom line?**

- Pulmonary injury in combination with enhanced B-cell activation is sufficient to induce pulmonary hypertension symptoms in mice and patients with IPAH show increased B-cell receptor signalling and increased circulating follicular T helper-17 polarisation, indicating that adaptive immune activation may contribute to vascular remodelling, disease induction or progression in IPAH.

**Why read on?**

- We believe that this novel perspective on IPAH aetiology will have relevance for the clinic, as it may open up new possibilities for therapy development.

## Background

Pulmonary arterial hypertension (PAH) is a fatal disease characterised by progressive pulmonary vascular remodelling mediated by endothelial cell dysfunction leading to increased pulmonary vascular pressure and right ventricle dysfunction.^1–3^

Idiopathic pulmonary arterial hypertension (IPAH) is a form of pulmonary hypertension (PH) in which no underlying disease or aetiology is identified at time of diagnosis. The presence of various immune cells in and around hypertensive pulmonary vascular lesions indicates their contribution to disease.^4 5^ Several lines of evidence point towards a role for increased adaptive immune responses and cytokines involved in vascular remodelling.^2 6^ It is currently unclear whether chronic immune activation is cause or consequence of the primary remodelling process.^7^ Nevertheless, accumulating evidence suggests that vascular and lung injury could lead to impaired tolerance to self-antigens, which may contribute to vascular remodelling and subsequent PH development.^2 8^ A considerable fraction of patients with IPAH has increased levels of circulating autoreactive plasmablasts and autoantibodies, which may often be produced by plasma cells in tertiary lymphoid structures in the lung.^9 10^

Well-regulated B-cell receptor (BCR) signalling is crucial to maintain self-tolerance.^11^ A key signalling protein downstream of the BCR is Bruton’s tyrosine kinase (BTK).^12^ Increased BTK levels are associated with active disease in several autoimmune disorders in humans.^13^ Moreover, B-cell-specific BTK overexpression in CD19-hBTK transgenic mice induced spontaneous germinal centre (GC) formation in the spleen, production of antinuclear autoantibodies and development of an autoimmune phenotype.^13 14^ Another crucial step for adequate B-cell activation and selection is T cell help from follicular T-helper (Tfh) cells.^15^ Tfh cells are antigen-experienced CD4^+^ T cells that express the B-cell follicle homing receptor CXCR5 and are therefore present in B-cell follicles in lymph nodes, spleen, Peyer’s patches and tertiary lymphoid organs (TLOs). A breakdown of self-tolerance could result from aberrant Tfh-cell activation in GCs.^16 17^ Interestingly, BTK overexpression in CD19-hBTK transgenic mice is associated with an increase in Tfh cells in the spleen and drives systemic autoimmunity by disrupting T-cell homeostasis, providing a detrimental feedforward loop.^18^ Tfh cells in TLOs and GCs are not easily accessible in patients. However, circulating CXCR5^+^CD4^+^ T cells, which are often referred to as circulating Tfh (cTfh) cells, share functional properties with Tfh cells.^19^ These cells are thought to represent a circulating pool of memory Tfh cells and alterations in Tfh cells are associated with autoimmunity in humans.^19 20^

Given the parallels of IPAH and autoimmune diseases, it is conceivable that aberrant B-cell activation and Tfh activity contribute to the aetiology of IPAH. Similar to our observation of robust induction of autoantibodies in the lung following influenza infection in CD19-hBTK mice,^13 14^ we hypothesised that autoantibodies reactive against pulmonary vascular structures may arise in these mice on induction of pulmonary injury. Although bleomycin exposure is often used to induce lung fibrosis in mice, several groups have applied the bleomycin mouse model to investigate experimental PH.^21 22^ Therefore, we investigated whether CD19-hBTK mice developed enhanced PH symptoms on bleomycin exposure. Next, we used 14-colour flowcytometry to investigate B cells and circulating Tfh-cell subsets and their activation status in peripheral blood of patients with IPAH. In these analyses, we also included blood samples from patients with connective tissue disease associated PAH (CTD-PAH), congenital heart disease PAH (CHD-PAH), interstitial lung disease related PH (ILD-PH) and healthy controls (HCs).

## Methods

### Subjects

Twenty-nine patients with PAH who entered the ‘Biomarker activity in adults with pulmonary hypertension’ (*BioPulse*) study or were diagnosed in the Erasmus MC (see table 1 for patient characteristics) were included in the study. Patients were diagnosed according to the current European Society of Cardiology (ESC) and European Respiratory Society (ERS) guidelines and therefore all patients underwent a right heart catheterisation.^23^ HCs (n=19) were age and sex matched. All patients and controls gave written informed consent. This study adheres to the Declaration of Helsinki and the Medical Ethical Committee of the Erasmus MC approved this study (MEC-2011-392 and 2012-512) and gave consent for collection of blood samples.

**Table 1:** Patient characteristics

	IPAH (n=13)*	CTD-PAH (n=9)†	CHD-PAH (n=7)‡
Gender (male)	3 (23%)	1 (11%)	5 (71%)
Age (years)	50 (33–60)	71 (50–74)	41 (26–40)
Time to diagnosis (months)	40 (12–80)	6 (3–15)	1 (1–12)
Right heart catheterisation			
mPAP (mm Hg)	58 (47–78)	37 (31–52)	43 (38–61)
RAP (mm Hg)	13 (8–19)	8 (6–11)	8 (6–9)
PAWP (mm Hg)	13 (11–17)	9 (5–13)	12 (10–16)
CI (L/min/m^2^)	2.4 (1.6–2.8)	3.1 (2.4–3.6)	3.3 (2.9–3.5)
CO (L/min)	4.2 (2.7–5.1)	5.6 (4.8–6.9)	6.0 (4.1–7.4)
PVR (dynes s/cm^5^)	1025 (628–1430)	371 (189–830)	426 (293–577)
SvO_2_ (%)	60 (56–73)	63 (61–75)	70 (60–79)
Functional parameters			
NYHA functional class 1 (n)	0 (0%)	0 (0%)	1 (14%)
NYHA functional class 2 (n)	5 (38%)	4 (44%)	2 (28%)
NYHA functional class 3 (n)	7 (54%)	4 (44%)	4 (57%)
NYHA functional class 4 (n)	1 (8%)	1 (11%)	0 (0%)
6MWD (m)	320 (226–365)	289 (267–432)	452 (263–592)
NT proBNP (μmol/L)	69 (32–203)	69 (15–79)	34 (11–43)
Vasoactive medication (n)**§**	10 (77%)	5 (56%)	0 (0%)

Healthy subject characteristics: n=19, gender (male) = 6 (32%), age (years) = 48 (30–52). Continuous variables are presented as median and IQR in parentheses and categorical variables as counts and percentages in parentheses.

*Genetic testing revealed that two patients had a *BMPR2* gene mutation.

†Seven patients with rheumatoid arthritis, one patient with systemic sclerosis, one patient with systemic lupus erythematosus.

‡Five patients with atrial septal defect, one patient with Eisenmenger’s syndrome and ventricular septal defect, one patient with major aortopulmonary collateral arteries.

§Vasoactive medication at the time when blood sample was taken.

CHD-PAH, congenital heart disease associated pulmonary arterial hypertension; CI, cardiac index; CO, cardiac output; CTD-PAH, connective tissue disease associated pulmonary arterial hypertension; IPAH, idiopathic pulmonary arterial hypertension; mPAP, mean pulmonary arterial pressure; 6MWD, 6 min walk distance; NYHA, New York Heart Association; PAWP, pulmonary arterial wedge pressure; PVR, pulmonary vascular resistance; RAP, right atrial pressure; SvO_2_, central mixed venous oxygen saturation.

### Flow cytometry procedures

Procedures of flow cytometry experiments are described in online supplemental methods.

10.1136/thoraxjnl-2020-215460.supp1 Supplementary data

### Self-reactive IgG

Plasma samples (1/50 diluted) of patients and HCs with PH were incubated for 1 hour on Kallestad HEp-2 slides (Bio-Rad Laboratories), using various Ig F(ab′)2 fragments as detection antibodies (online supplemental table 1). Fluorescence intensities of HEp2 slides signals were evaluated using an LSM 311 META confocal fluorescence microscope (Zeiss) and LSM Image Browser V.4.2.0.12 software (Zeiss).

### Mice

CD19-hBtk transgenic mice^13^ were bred on the C57BL/6J background and kept under specified pathogen-free conditions in the Erasmus MC experimental animal facility. Experimental protocols were reviewed and approved by the Erasmus Medical Center MC Committee of animal experiments. Procedures of mouse experiments are described in online supplemental methods.

### Principal component analysis

Principal component analyses (PCA) were performed using R and RStudio, and the packages FactoMineR and Factoextra. Prior to PCA, data were log10-transformed to better fit a normal distribution and scaled. Contribution of the variables to the dimensions was determined in percentages by (squared cosine of the variable × 100)/ (total squared cosine of the principal component).

### Statistical analyses

For calculating the significance of differences between >2 groups, we used the Kruskal-Wallis test combined with a Dunn’s multiple comparison test. Mann-Whitney U test was used for comparison of two groups. The variability explained by the PCA was tested for statistical significance by inertia of the first two dimension using the R package FactoInvestigate. Correlation coefficients were calculated using Spearman’s rank method. Statistical analyses were performed using IBM SPSS Statistics V.21 and GraphPad Prism V.6 software. P values<0.05 were considered significant.

## Results

### CD19-hBTK transgenic mice develop PH and local autoreactivity on bleomycin exposure

**Figure 1:**
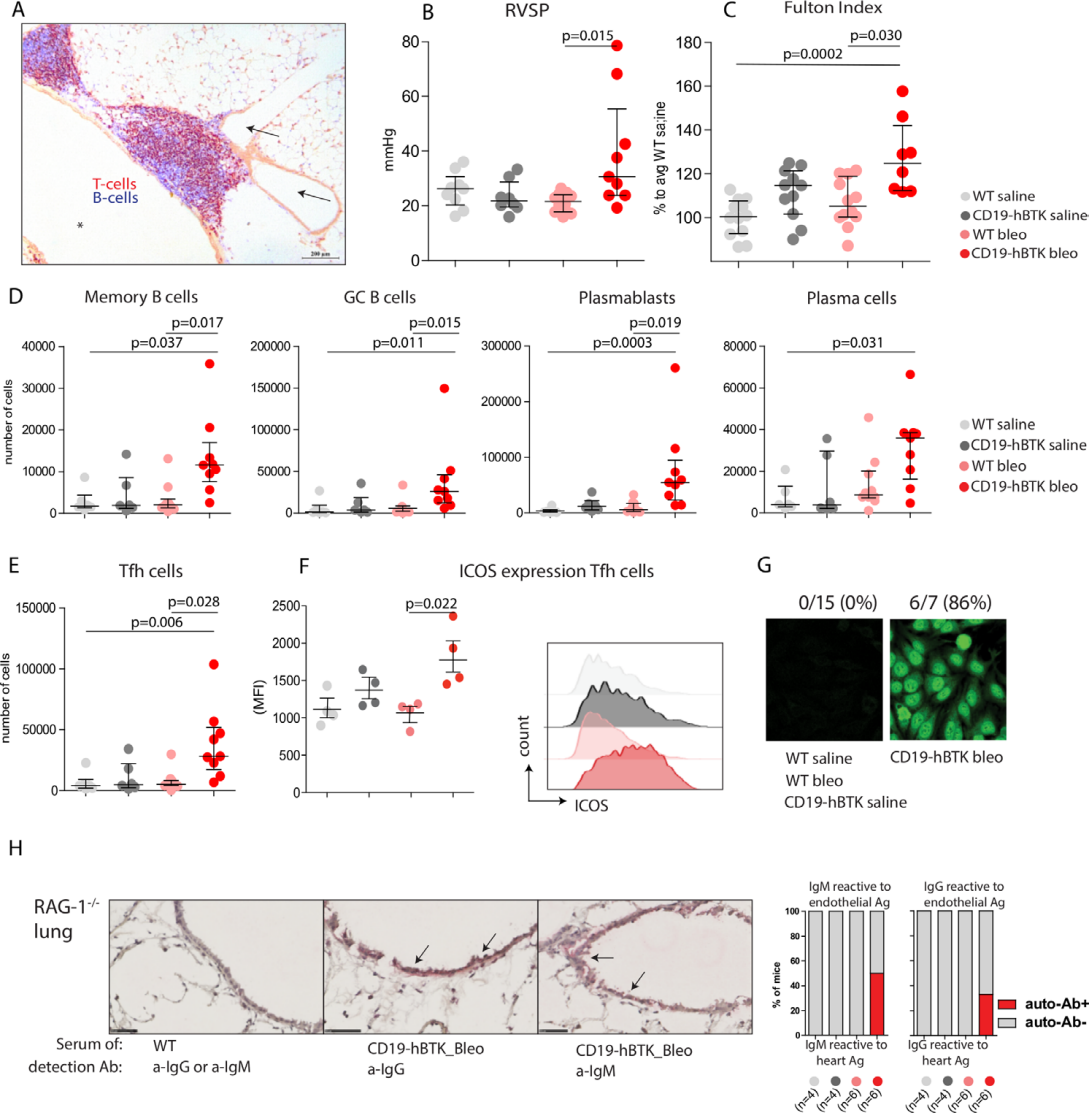
CD19-hBTK transgenic mice develop pH and local autoreactivity on bleomycin exposure. (A) Pulmonary inflammatory infiltrate in lung tissue (cryo-section) in a 40-week-old CD19-hBTK mouse stained with anti-CD3 (T cells, *red*) and anti-B220 (B cells, *blue*). This inflammatory infiltrate is located next to a pulmonary vessel (*arrows*) and airway (*asterisk*). (B–C) Pulmonary hypertension indices in WT and CD19-hBTK mice 10 weeks after saline or bleomycin exposure: RVSP (B) and Fulton index (ratio of right ventricular to left ventricular and septal weight) (C) in the indicated four mouse groups. (D) Quantification of total numbers of B-cell subpopulations in WT and CD19-hBTK transgenic mice 10 weeks after bleomycin or saline exposure: memory B cells (CD19^+^B220^+^CD80^+^PDL2^+^), GC B cells (CD19^+^B220^+^IgD^-^CD95^+^), plasmablasts (CD19^+^CD138^+^) and plasma cells (CD19^low^CD138^+^). (E) Total numbers of Tfh cells (CD3^+^CD4^+^CXCR5^+^PD1^+^) in MedLN. (F) MFI values of ICOS expression on gated MedLN Tfh cells (*left*) and representative histogram overlays of ICOS expression on gated Tfh cells (*right*) (G) representative staining patterns of human epithelial cells (HEp)-2 slides with serum of the indicated mice WT and CD19-hBTK mice, at 3 weeks after bleomycin or saline exposure. (H) Cryosections of lung tissue of *Rag1^−/−^* mice incubated with serum of WT or CD19-hBTK mice, 10 weeks after bleomycin or saline exposure, as indicated. Anti-IgM or anti-IgG was used for visualisation of antibodies reactive to endothelial antigens (*arrows*) (*left*). Proportions of mice in which antibodies reactive to lung antigens were detected in the serum (*right*). The survival rates for the four mouse groups at ~10 weeks were: 100% for saline treated mice, 83% for bleomycin/WT and 65% for bleomycin/CD19-hBTK mice. The results are shown as median (IQR), p exact values were obtained following a Kruskal-Wallis test (B–F). Dots represent individual mice. GC, germinal centre; ICOS, inducible costimulator; MFI, mean fluorescence intensity; RVSP, right ventricular systolic pressure; Tfh, follicular T-helper; WT, wildtype.

We have previously shown that aged CD19-hBTK transgenic mice spontaneously develop autoimmune pathology, accompanied by inflammatory infiltrates around pulmonary vascular structures with segregated T-cell and B-cell zones (figure 1A).^13^ To provoke lung injury, we subjected these mice to intratracheal bleomycin (or saline as a control). Because the bleomycin model is generally employed as a model for lung fibrosis, we first investigated whether the presence of the CD19-hBTK transgene would lead to augmented fibrosis at 3 and 10 weeks after bleomycin exposure (online supplemental figure 1). The hydroxyproline content, lung tissue elastance and total fibrosis score were similar between hBTK-mice and control littermates at 3 weeks and a similar degree of resolution of fibrosis occurred at 10 weeks, as previously reported for wild-type (WT) mice.^24^

PH symptoms have been reported at early timepoints (2–4 weeks) after bleomycin exposure in mice.^21 22^ At 10 weeks after exposure, we still observed a significant bleomycin-induced increase of right ventricular systolic pressure (RVSP, p=0.015) and signs of right ventricular hypertrophy (Fulton Index, p=0.030) in CD19-hBTK mice, but not in WT mice (figure 1B, C). In both mouse strains the numbers of pulmonary vessels with perivascular and endothelial cells expressing α-smooth muscle actin (α-SMA) and the proportions of α-SMA-positive areas of the total artery did not significantly change at 10 weeks after bleomycin exposure (online supplemental figure 2).

To examine whether lymphocyte activation on bleomycin exposure was enhanced in CD19-hBTK mice, we analysed the B-cell compartment in mediastinal lymph nodes (MedLNs) using the gating strategy in online supplemental figure 3. The numbers of memory B cells (CD19^+^B220^+^CD80^+^PDL2^+^), GC B cells (CD19^+^B220^+^IgD^-^CD95^+^), plasmablasts (CD19^+^CD138^+^) and plasma cells (CD19^low^CD138^+^) in MedLNs of CD19-hBTK mice exposed to bleomycin were significantly increased (p=0.017, p=0.015, p=0.019, p=0.031, respectively), compared with bleomycin-exposed WT mice (figure 1D). Likewise, MedLN from bleomycin-exposed CD19-hBTK mice showed a significant increase (p=0.028) of Tfh-cells (figure 1E), which displayed enhanced surface expression of inducible costimulator (ICOS, p=0.022), a marker for T-cell activation (figure 1F).

Next, we evaluated whether increased B-cell activation via BTK upregulation also resulted in increased autoantibody production. At 21 days post exposure ~86% of bleomycin-treated CD19-hBTK mice—and none of the control littermates—had serum antinuclear IgG autoantibodies, as shown by HEp2 reactivity (figure 1G), but at day 70 these autoantibodies were no longer detectable. At 70 days post exposure, serum of a fraction of bleomycin-treated CD19-hBTK mice—and not any of the other mice—was positive for autoreactive IgM (~50%) and IgG (~33%) recognising endothelial antigens, as detected in cryosections of lung tissue of *Rag1^−/−^* mice (figure 1H).

In conclusion, CD19-hBTK mice developed haemodynamic and cardiac signs of PH after pulmonary injury with bleomycin, which was not seen in WT mice. Additionally, bleomycin-induced pulmonary injury in CD19-hBTK mice resulted in enhanced B-cell activation and production of autoantibodies, including antibodies recognising vascular structures in the lung. Although we did not observe profound vascular remodelling in this model, these findings were hypothesis-generating and suggested that increased B-cell activation may enhance the susceptibility to PH development.

### Decreased proportions of circulating non-switched memory (NSM) B cells in patients with PAH

Given the link between BTK expression, B-cell activation and PH development in mice, we evaluated B-cell subsets and BTK expression in peripheral blood of patients with IPAH (n=13) and compared these with HCs (n=19) and patients with CTD-PAH (n=9) or CHD-PAH (n=7). Increased B-cell activation and autoantibody reactivity against vascular structures contribute to the development of CTD-PAH and therefore this subgroup served as a ‘positive’ disease control.^25^ CHD-PAH is mainly driven by (congenital) structural cardiac changes and served as a ‘negative’ disease control. Patient and HC characteristics are shown in table 1.

**Figure 2:**
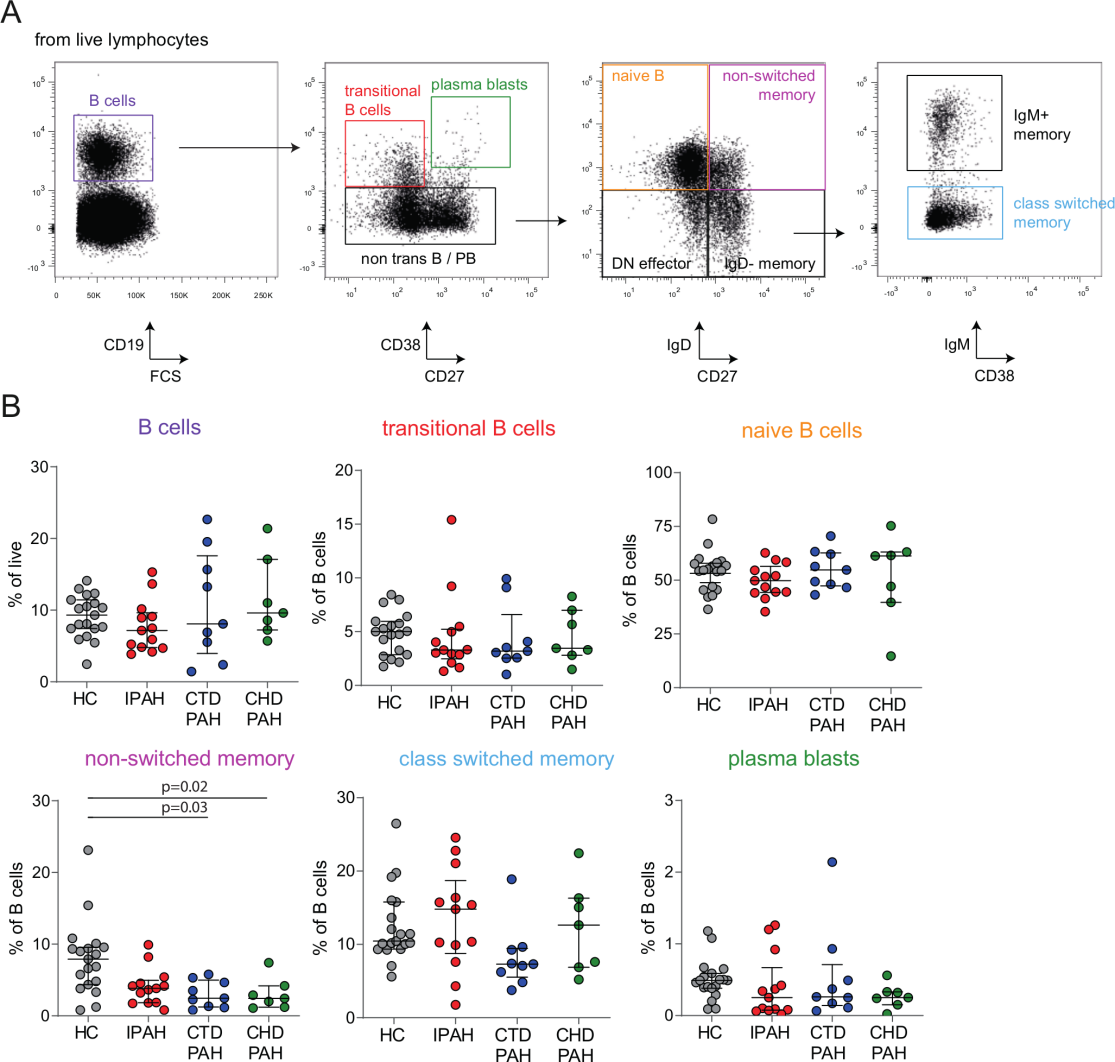
Decreased proportions of circulating NSM B cells in patients with PAH. (A) Representative gating strategy for the identification of B-cell subsets in peripheral blood. Starting with live single cells, total B cells (CD19^+^), transitional B cells (CD19^+^CD38^+^CD27^−^), plasmablasts (CD19^+^CD38^hi^CD27^+^), naive B cells (CD19^+^IgD^+^CD27^−^), NSM B cells (CD19^+^IgD^+^CD27^+^), IgD^−^ memory B cells (CD19^+^IgD^−^CD27^+^), IgM^+^ memory B cells (CD19^+^CD27^+^IgD^−^IgM^+^) and switched memory B cells (CD19^+^CD27^+^IgD^−^IgM^−^) were identified. (B) Proportions of circulating total B cells and the indicated B-cell subpopulations in HC (n=19) and patients with IPAH (n=13), CTD-PAH (n=9) and CHD-PAH (n=7). The results are shown as median (IQR), p exact values were obtained following Kruskal-Wallis test. Dots represent values in individual patients or HC. CHD-PAH, congenital heart disease pulmonary arterial hypertension; CTD-PAH, connective tissue disease-associated pulmonary arterial hypertension; FCS, forward scatter; HC, healthy controls; IPAH, idiopathic pulmonary arterial hypertension; NSM, non-switched memory.

We used flow cytometry to quantify total peripheral blood CD19^+^ B cells, as well as CD38^+^CD27^−^ transitional, IgD^+^CD27^−^ naive, IgD^+^CD27^+^ and IgD^−^IgM^+^CD27^+^ NSM, IgD^−^IgM^−^CD27^+^ switched memory B cells and CD38^+^CD27^+^ plasmablasts, following the gating strategy in figure 2A. The total proportions of B cells did not differ between patients and control groups (figure 2B) with IPAH. Within the B-cell population, NSM B cells were decreased in all three PAH subgroups compared with HC, reaching significance for patients with CTD-PAH (p=0.03) and patients with CHD-PAH (p=0.02), paralleling published findings in rheumatoid arthritis and systemic sclerosis.^26 27^

### Increased BTK protein expression and phosphorylation in circulating B cells of patients with IPAH

Because increased BTK levels in B cells were associated with enhanced PH development in mice, we next evaluated BTK expression and BCR signalling in circulating B cells in the three groups of patients and HCs with PAH.

**Figure 3:**
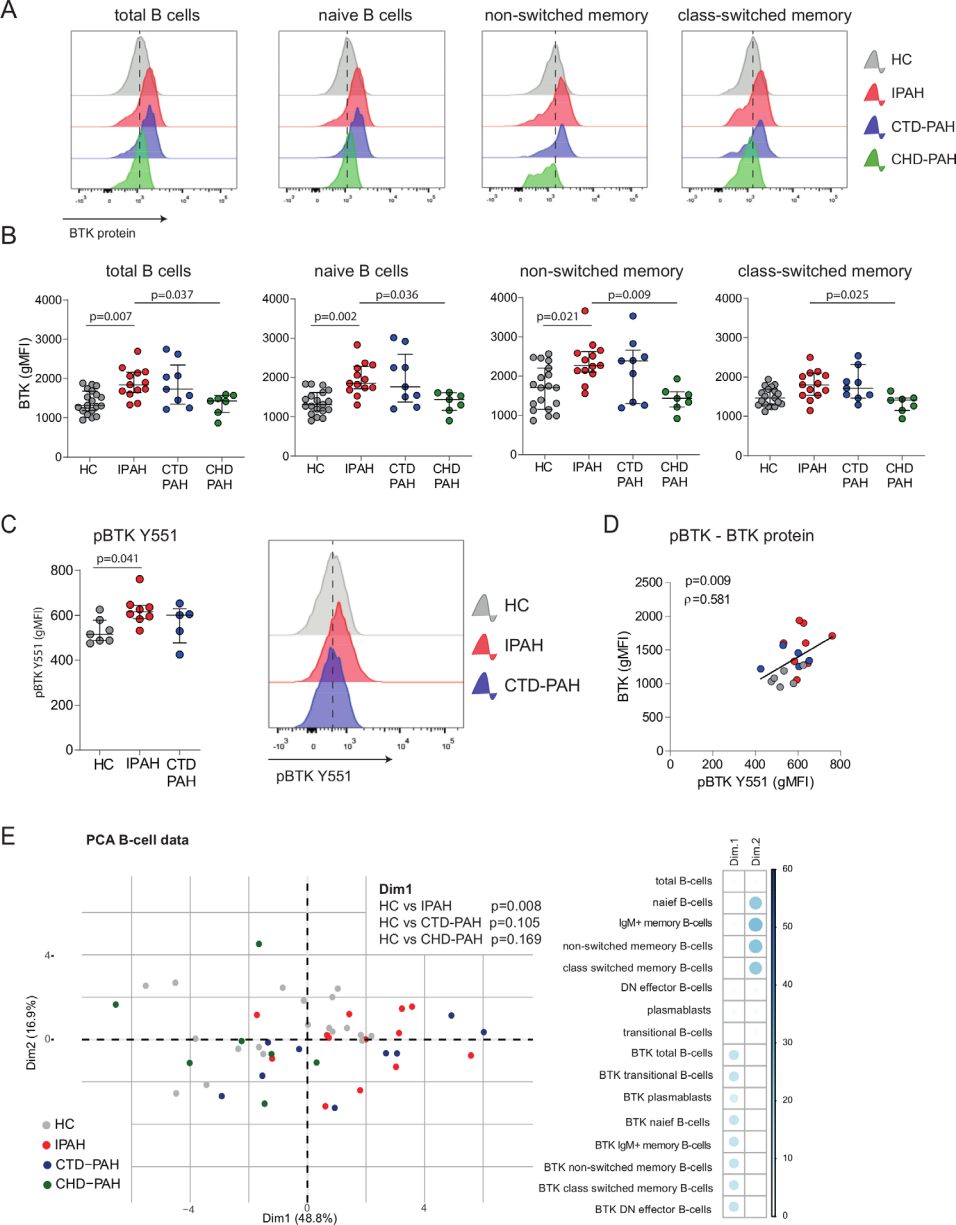
Increased BTK protein expression and BCR signalling in circulating B cells of patients with IPAH. (A) Representative histogram overlays of BTK geometric mean fluorescence intensity (gMFI) values assessed by intracellular flow cytometry analysis of the indicated B-cell subpopulations of HCs and patients with IPAH, CTD-PAH and CHD-PAH, as indicated. (B) Quantification of BTK protein expression levels, shown as gMFI values of intracellular flow cytometry analysis of the indicated B-cell subpopulations in HCs and the three patient groups. (C) Quantification of phosphorylated BTK protein (pBTK-Y551) levels, shown as gMFI values of intracellular flow cytometry analysis (*left*) and representative histogram overlays of pBTK-Y551 expression (*right*). (D) Correlation of pBTK and BTK protein expression in HCs and patients with IPAH and CTD-PAH. Correlation coefficient was calculated using Spearman’s rank method. (E) PCA of B-cell subsets and BTK levels in PH subgroups and HC. Contribution of variables on the first dimension (Dim1) and second dimension (Dim2) of the PCA. The PCA of the B-cell subsets and BTK levels showed a non-random distribution over Dim1 and Dim2, which was not due to gender (p=0.083 (Dim1) and p=0.378 (Dim2)) or age (p=0.069 (Dim1) and p=0.790 (Dim2)). PCA were on log10-transformed and scaled values. The results are shown as median (IQR; B–D), p exact values were obtained following Kruskal-Wallis test (>2 groups) or Mann-Whitney U test. dots represent values in individual patients or HC (B–E). BTK, Bruton’s tyrosine kinase; CTD-PAH, connective tissue disease associated pulmonary arterial hypertension; CHD-PAH, congenital heart disease pulmonary arterial hypertension; gMFI, geometric mean fluorescence intensity; HCs, healthy controls; IPAH; idiopathic pulmonary arterial hypertension; PCA, principal component analyses; PH, pulmonary hypertension.

Representative histogram overlays showing BTK protein expression in total B cells and B-cell subsets, as determined by intracellular flow cytometry, are depicted in figure 3A. We observed higher BTK protein levels in all B-cell subsets, except for class-switched memory B cells, in patients with IPAH and CTD-PAH compared with HCs and patients with CHD-PAH (figure 3A; quantified in figure 3B).

To link BTK protein levels to its activity, we measured BTK phosphorylation at Y551 (pBTK) by phosphoflow analysis in B cells from patients ex vivo (without in vitro stimulation). pBTK expression was significantly increased (p=0.041) in B cells from patients with IPAH compared with HCs (figure 3C) with IPAH and correlated significantly (p=0.009) with BTK protein levels (figure 3D).

To obtain a more comprehensive overview of the B-cell profiles across the three patient groups and HCs, we performed a principal component analysis (PCA) of 16 parameters of B-cell subsets and BTK expression levels. HCs and patients with IPAH were significantly separated by dimension 1 (Dim1), to which particularly BTK levels across B-cell subsets contributed (p=0.008; figure 3E) and not proportions of B-cell subpopulations, which dominated Dim2.

Our finding that bleomycin as a second trigger induced PH in the CD19-hBTK mouse model, raised the question if BTK expression is also increased in ILD-PH. Analysis of a cohort of patients with ILD-PH (see for patient characteristics: online supplemental figure 4A) did not reveal alterations in B-cell subsets, compared with HCs (online supplemental figure 4B). We even observed decreased BTK protein levels in B cells in patients with ILD-PH, compared with HC, which reached significance in patients receiving treatment (p=0.001; online supplemental figure 4C). This analysis suggests that enhanced B-cell activation is not a crucial driver of ILD-PH.

Taken together, our multivariate data analysis demonstrates that BTK protein expression was increased in circulating B cells of patients with IPAH. This increase in BTK protein also reflected augmented BCR signalling.

### Autoreactive IgG antibodies in plasma of patients with IPAH correlate with BTK levels

**Figure 4:**
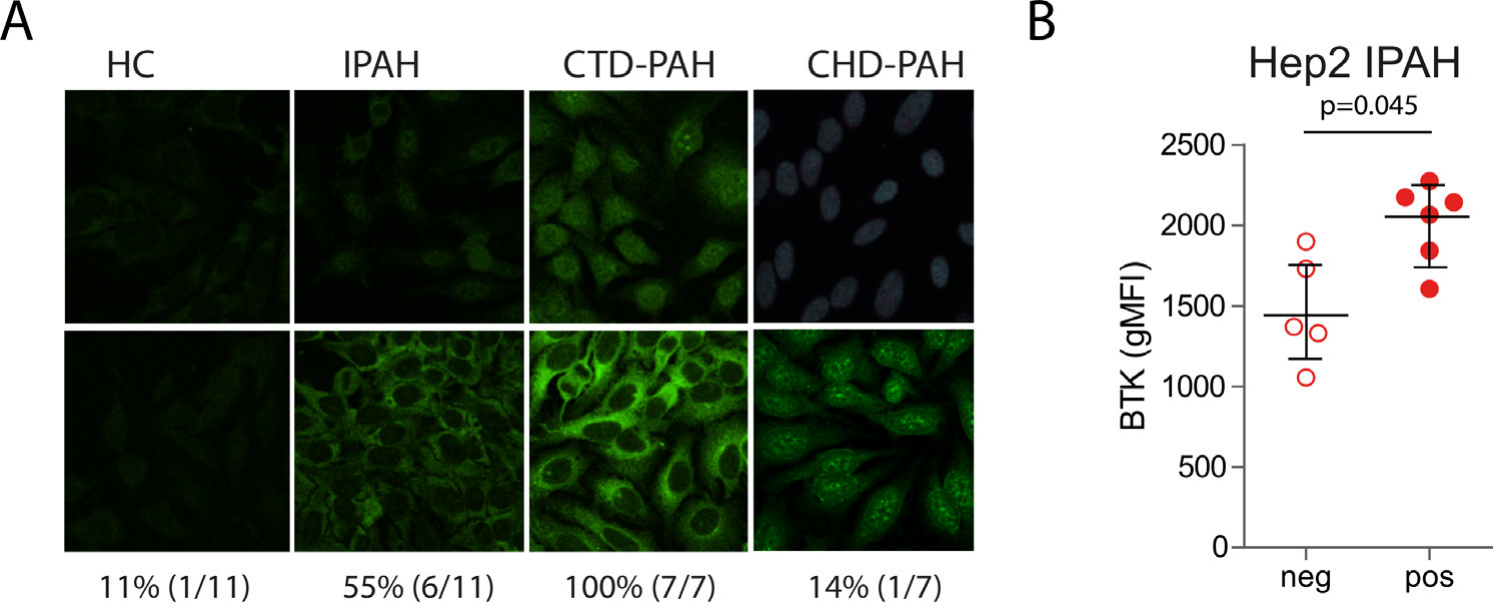
Autoreactive IgG antibodies in plasma of patients with IPAH correlate with BTK levels. (A) Staining pattern of human epithelial cell (Hep)-2 slides incubated with plasma of HCs and patients with IPAH, CTD-PAH and CHD-PAH. As detection antibodies IgG F(ab′)2 fragments were applied. For each group two representative slides are depicted. (B) Quantification of BTK protein expression levels (gMFI values) in total circulating B cells from patients who were Hep2-positive and Hep2-negative and with IPAH. The results are shown as median (IQR), p exact values were obtained following Mann-Whitney U test. Dots represent values in individual patients. BTK, Bruton’s tyrosine kinase; CTD-PAH, connective tissue disease associated pulmonary arterial hypertension; CHD-PAH, congenital heart disease pulmonary arterial hypertension; gMFI, geometric mean fluorescence intensity; HCs, healthy controls; IPAH; idiopathic pulmonary arterial hypertension.

To explore the presence of autoantibodies in plasma of patients and controls with PH, we screened HEp-2 slides (figure 4A). Six (~55%) out of 11 patients with IPAH had detectable autoreactive IgG, versus 1 of 11 HCs and 1 of 7 patients with CHD-PAH. As expected, all CTD-PAH had detectable IgG autoantibodies. Interestingly, the patients with IPAH who were HEp2-positive had higher intracellular BTK protein levels compared with the patients with IPAH who were HEp2-negative, p=0.045 (figure 4B).

This finding indicates that increased intracellular BTK protein expression in peripheral blood B cells of patients with IPAH was associated with the presence of circulating autoantibodies.

### Increased proportions of cTfh-17 cells in patients with IPAH

**Figure 5:**
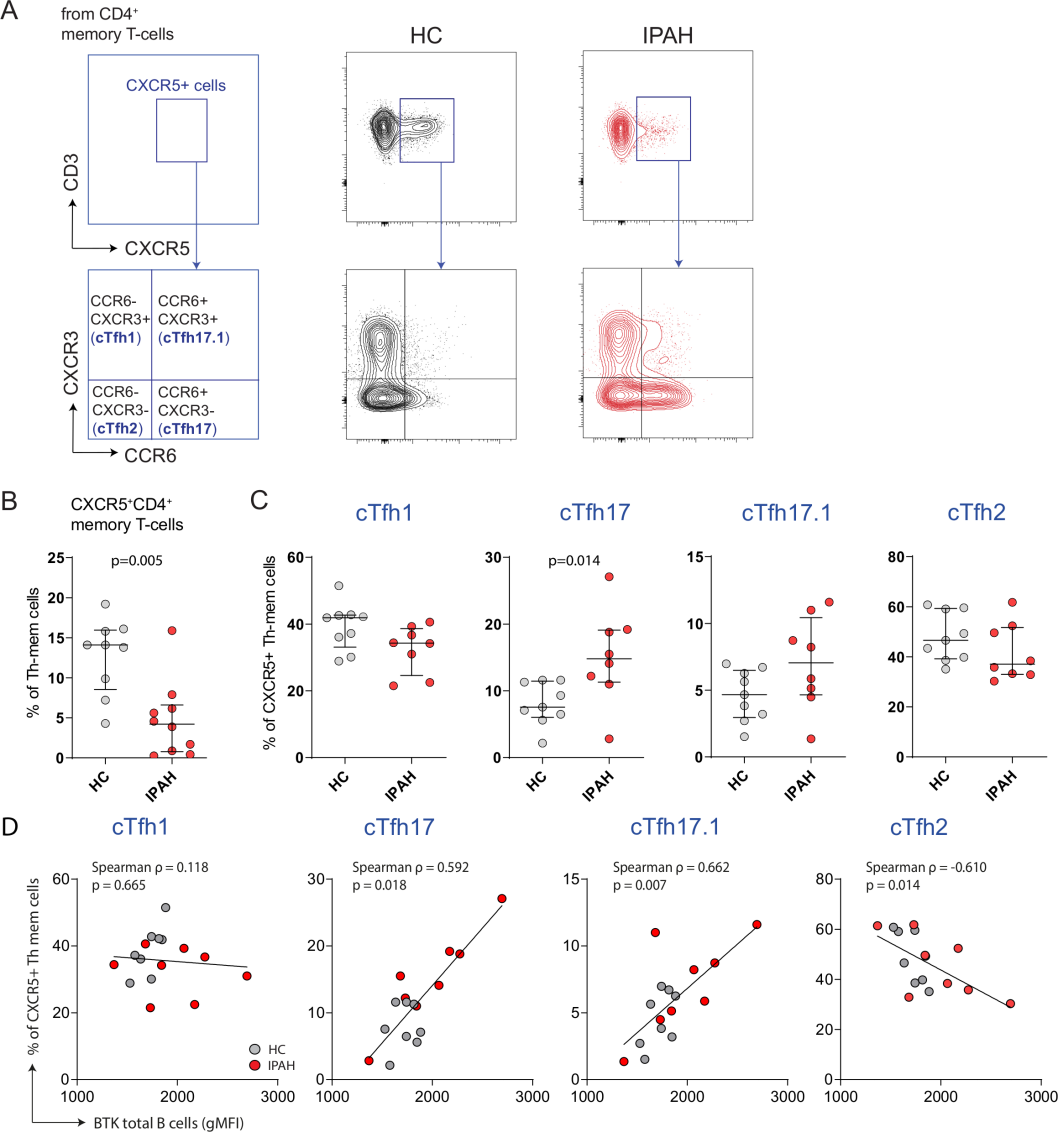
Increased proportions of cTfh-17 cells in patients with IPAH. (A) Representative gating strategy for the identification of CXCR5^+^ cTfh cells, starting from non-regulatory (CD25^+^CD127^low^) CD4^+^CD45RA^−^ memory T cells, and further discrimination of cTfh subsets based on surface CCR6 and CXCR3 expression: cTfh-1 (CCR6^−^CXCR3^+^), cTfh17.1 (CCR6^+^CXCR3^+^), cTfh17 (CCR6^+^CXCR3^−^) and cTfh2 (CCR6^−^CXCR3^−^), as depicted for a HC and patient with IPAH. (B) Proportions of CXCR5^+^ memory T cells as percentages of total memory CD4^+^ T cells. (C) Proportions of the indicated cTfh subsets as percentages of CXCR5^+^ memory CD4^+^ T cells. (D) Correlations of cTfh subsets and BTK protein in total B cells. The results are shown as median (IQR), p exact values were obtained following Mann-Whitney U test. Correlation coefficient was calculated using Spearman’s rank method. Dots represent values in individual patients. BTK, Bruton’s tyrosine kinase; cTfh, circulating follicular T helper; IPAH, idiopathic pulmonary arterial hypertension.

As we found BTK overexpression in B cells from patients with IPAH, we focused on this group of patientsH for the evaluation of cTfh cells, which can be induced by BTK overexpression in B cells.^18^ Based on surface C–C chemokine receptor 6 (CCR6) and C–X–C motif chemokine receptor 3 (CXCR3) expression, different cTfh subsets can be discriminated with cytokine profile characteristics of Th1, Th2 and Th17 cells^19^ (figure 5A).

Patients with IPAH exhibited significantly reduced proportions of CXCR5^+^ memory T cells in the circulation (p=0.005; figure 5B). Within this cell population, we noticed an altered cTfh subset distribution. The fraction of cTfh17 cells was significantly increased (p=0.014) compared with HCs (figure 5C). Interestingly, in HCs, and patients with IPAH increased BTK protein levels in B cells correlated with increased proportions of circulating cTfh17 (p=0.018) and cTfh17.1 cells (p=0.007). BTK protein levels in B cells showed a negative correlation with proportions of cTfh2 cells (p=0.014; figure 5D) and did not correlate with proportions of cTfh1 cells (figure 5D) or total cTfh cells (online supplemental figure 5).

In conclusion, patients with IPAH displayed an imbalance in cTfh-cell subset distribution, with increased cTfh17 cells, whereby increased cTfh17 and cTfh 17.1 proportions correlate with enhanced B-cell activation.

## Discussion

To investigate the involvement of activated B cells in the pathogenesis of IPAH, we studied an autoimmune-prone mouse model with enhanced BCR signalling, as well as circulating B cells and T cells in three groups of patients with PAH.

We observed that CD19-hBTK transgenic mice with increased protein levels of the BCR-associated kinase BTK specifically in B cells developed haemodynamic and cardiac signs of PH, following induction of pulmonary injury with bleomycin. In this hypothesis-generating two-hit model, the MedLNs contained active GCs with prolonged B-cell activation and increased proportions of activated ICOS^high^ Tfh cells, while autoantibodies with reactivity against vascular antigens were present in the serum. In parallel, peripheral blood B cells from patients with IPAH displayed increased BTK protein expression, already in naive B cells. The increase in BTK levels was associated with enhanced BTK phosphorylation in B cells, the presence of IgG autoantibodies in plasma, as well as higher proportions of circulating Tfh17 cells. Taken together, our study provides evidence that loss of immune homeostasis—characterised by altered BCR signalling in (naive) B cells, activation of B cells in GCs and an unbalanced Tfh-cell subset distribution—can contribute to PH development.

Our findings are in line with RNA expression studies on peripheral blood total B-cell samples that suggest that B cells are activated in patients with IPAH, compared with HCs.^28^ Several checkpoints exist in the bone marrow and in the periphery that prevent the development of autoreactive B cells and their inadvertent activation. However, enhanced BCR signalling due to BTK overexpression in mice is sufficient to induce resistance to Fas-mediated apoptosis and development of B-cell mediated autoimmunity.^13^ Parallel to our recent observation of increased BTK expression and phosphorylation in patients with anticitrullinated protein antibody-positive rheumatoid arthritis, Sjogren’s syndrome and autoimmune vasculitis,^14 29^ we found that BTK expression was also increased in naive B cells in patients with IPAH. Since such B cells have not encountered antigen, a possible explanation of these findings is that signals from the microenvironment, such as cytokine and chemokine levels or presence of Toll-Like receptor ligands, induce changes in the epigenome, transcriptome or proteome of naive B cells.^30^ It is therefore attractive to speculate that the threshold for activation of naive B cells by self-antigens is reduced, which would then contribute to engagement of naive B cells in IPAH pathology. Alternatively, it is possible that in patients with IPAH the proportions of circulating autoreactive naive B cells is increased as a result of defective self-tolerance in developing B cells, as was also shown in the autoimmune disease systemic lupus erythematosus.^31^ In any case, we found that 6 out of 11 patients with IPAH had circulating autoantibodies as well as high BTK levels in their peripheral blood B cells. We therefore conclude that in this regard the phenotype of patients with IPAH is most probably heterogeneous, whereby a substantial fraction of patients with IPAH displays striking immunological similarities to patients with CTD-PAH.^32^

We observed that BTK levels were not increased in untreated patients with ILD-PH and were decreased in patients receiving treatment with antifibrotics. Although the pathogenesis of ILD-PH is multifactorial, sustained hypoxia is believed to be one of the most frequent inducers of PH in this group.^33 34^ Chronic hypoxia may lead to decreased nitric oxide, increased hydrogen peroxide (H_2_O_2_, and alterations in voltage-gated K^+^ channels.^33 34^ It is conceivable that these mechanisms leading to hypoxic-induced PH operate in an inflammation-independent way and do not involve enhanced B-cell activation. Because BCR signalling is sensitive to treatment or disease remission,^14 29^ antifibrotic treatment of patients wit ILD-PH might have influenced BTK protein expression.

We observed that in patients with PAH, the proportions of NSM B cells in the circulation were decreased, which was also observed in various autoimmune diseases.^26 27 35^ This is particularly interesting because self-reactive B cells are removed from the repertoire at the transition from CD27^−^ naive B cell to non-switched CD27^+^ memory B cell, thus at a stage that precedes the induction of somatic hypermutation.^36^ These NSM B cells have the capacity to interact with T cells in secondary and tertiary lymphoid organs in patients with IPAH, whereby their increased BTK expression levels may disrupt T-cell homeostasis. This would be supported by our finding that increased BTK-mediated signalling in B cells involves a positive-feedback loop that establishes T-cell-propagated autoimmune pathology and is accompanied by increased proportions of splenic Tfh cells.^18^ Hereby, the increased surface expression of the costimulatory molecules CD80 and CD86 on CD19-hBTK transgenic B cells likely supports T-cell activation. Interestingly, BTK levels in B cells correlated with ICOS expression on cTfh cells in patients with rheumatoid arthritis and with parotid gland T-cell infiltration in patients with Sjögren syndrome.^14^ In this context, it is of note that we found that proportions of cTfh17 cells were increased in patients with IPAH, similar to findings in systemic sclerosis^20^ and correlated with BTK expression levels in circulating B cells. In general, IL-17 immune polarisation is a feature of several chronic inflammatory and autoimmune conditions.^37^ Moreover, mounting evidence suggests that Th17 immune polarisation is also a feature of PAH. CD4^+^ T cells isolated from patients with PAH contain higher levels of IL-17A.^38 39^ Additionally, the numbers of Th17 cells are increased and these cells are often localised in perivascular regions.^6 38 39^ Also adoptive transfers of Th17 cells to *Rag1^−/−^* mice, lacking mature T cells and B cells, induced PH symptoms independent of chronic hypoxia.^38^ However, whether the cTfh17 or cTfh17.1 cells represent a functional and clinically relevant T-cell subtype is currently unknown and is a topic for further research.

The use of a bleomycin mouse model for PH research raises some concerns, since bleomycin exposure induces extensive lung damage, fibrotic changes and PH symptoms at 3 weeks.^21 22 40^ It is unclear if PH indices persist at later time points, but resolution of fibrosis occurs before ~10 weeks after bleomycin exposure.^24^ Although endothelial damage or pulmonary injury associated with viral infections are thought to be involved in PAH development,^41^ the extent and dynamics of lung damage in the bleomycin mouse model generally does not reflect lung injury seen in patients. Moreover, the bleomycin mouse model depends on inflammation and therefore we cannot formally rule out that additional inflammatory processes critically contribute to PH development in our mouse model. It is conceivable that PH development in CD19-hBTK mice may be promoted by chronic B-cell activation and autoantibodies. This would be supported by our observation that influenza virus infection provoked rapid autoantibody formation in the lungs of CD19-hBTK mice.^13^ Nevertheless, it would be interesting to explore if enhanced BCR signalling in other PAH models, such as the BMPR2-targeted mice, would lead to progressive PAH. Although our mouse model is primarily hypothesis-generating, we nevertheless observed interesting similarities with human B-cell pathobiology.

Together with the described anomalies in the B-cell compartment, suggestive for a sustained immune response against self-antigens in patients with PAH,^9 10 28 32 42^ our findings indicate that B-cell modulating therapies may hold potential for the treatment of PAH. Accordingly, B-cell depletion with anti-CD20 antibodies attenuated PH development in rats exposed to SU5416 and ovalbumin immunisation.^43^ Very recently, a double-blinded, randomised placebo-controlled clinical trial including 57 patients with systemic sclerosis (SSc)-PAH indicated that B-cell depletion therapy with anti-CD20 antibody rituximab is a potentially effective and safe adjuvant treatment for SSc-PAH.^44^ Whether anti-B-cell therapies could be effective in patients with IPAH needs to be further evaluated. Crucial will be the selection of patients with IPAH eligible for B-cell modulating therapies next to the current standard of care with vasoactive medication. Selecting patients should be based on inflammatory biomarkers, preferably those that reflect B-cell (auto-)reactivity or activation status. From our perspective, BTK levels could be a candidate biomarker, however its usefulness should be tested in prospective trials.

A limitation of our study is that we investigated only a relatively small number of patients within each PAH WHO subclass, which precluded subgroup analyses and identification of correlations with clinical parameters or survival. Further research with larger sample sizes is needed to confirm our findings.

In conclusion, our findings provide evidence that enhanced BCR signalling in B cells and increased circulating Tfh17 cell polarisation—together with lung damage—contribute to autoimmune-mediated vascular remodelling and disease pathogenesis in patients with IPAH. This new perspective on the aetiology of IPAH may prompt future translational studies on immune cells and inflammatory mediators as a potential diagnostic or prognostic marker in IPAH. Moreover, the identification of loss of immune homeostasis in patients with IPAH points to potential for new therapeutic anti-inflammatory targets in selected patients, next to existing vasoactive therapies.

## Data Availability

Data are available upon reasonable request.
