# Supplementary material for: Loss of immune homeostasis in patients with idiopathic pulmonary arterial hypertension
Source: Thorax. 2021 May 7;76(12):1209–18. doi: 10.1136/thoraxjnl-2020-215460 (PMC8606455; doi:10.1136/thoraxjnl-2020-215460)
Supplement: Supplementary data [file thoraxjnl-2020-215460supp001.pdf]

Online Supplementary data to:

Loss of Immune Homeostasis in Patients with Idiopathic Pulmonary Arterial Hypertension

Peter Heukels et al

online supplementary methods

Human flow cytometry procedures

Blood samples were collected in ethylenediaminetetraacetic acid (EDTA) tubes (BD Vacutainer K2E). Peripheral blood mononuclear cells (PBMCs) and plasma were obtained, processed and stored according to standard protocols.[1]

PBMCs were stained for extra- and intracellular markers (**online supplementary table 1**). To prevent non-specific labeling Fc-block (Anti-Mouse CD16/CD32 Fc-Block) was used. Fixable Viability Dye eFluor 506 (eBiosciences) was applied as a live-dead marker. Flow cytometry procedures for B-cell staining have been described previously[2]. Cells for the T-cell staining were first incubated in MACS buffer (0.5% BSA + 2mM EDTA in PBS) with fluorescent antibodies against chemokine receptors for 60 minutes at 4°C. A second extracellular incubation step was performed for antibodies with Brilliant Violet (BV) conjugates in Brilliant Stain-buffer (BD Biosciences, cat#563794). After fixation and permeabilization, cells were incubated with a forkhead box P3 (FOXP3)-specific antibody in permeabilization buffer for 60 minutes at 4°C. Live cells (>200,000) were acquired and data were analyzed by FACS Flow-Jo software.

mouse experiments and procedures

Genotyping and inducing pulmonary injury

CD19-hBtk[3] on a mixed background (Fvb × 129/Sv × C57BL/6J) were backcrossed on C57BL/6J for > 10 generations. Genotyping was performed by polymerase chain reaction (PCR), as previously described.[4] Wild-type mice used for the experiments are non-transgenic littermates. Mice were bred and kept at specified pathogen-free conditions in the Erasmus MC experimental animal facility. All experimental protocols have been reviewed and approved by the Erasmus MC Committee of animal experiments.
To induce pulmonary injury, bleomycin-hydrochloride was administered intra tracheally in 8-10 week old mice (0,04U/80 µl saline) or saline as a control as previously described.[5] Mice were sacrificed 21 days and 70 days after bleomycin exposure.

Right ventricular systolic pressure (RVSP) and lung tissue elastance

Mice were anaesthetized with isoflurane and right ventricular pressures were recorded using right heart catheterizations (mikro-tip catheter 1,4F , Millar instruments, model SPR-671) and analyzed with WinDaq Data acquisition software. Lung tissue elastance was measured with a flexiVent FX system as previously described.[6] Data was analyzed with flexiWare 7 software.

Flow cytometric procedures

Preparations of single-cell suspensions of MLN using standard procedures. Monoclonal antibodies are listed in **online supplementary table 1**. For intracellular staining, cells were fixed in Cytofix/Cytoperm and permeabilized, and then stained in Perm/Wash buffer (BD Bioscience). All measurements were performed on a LSRII flow cytometer (BD Bioscience), and results were analyzed using FlowJo software.

Immunohistochemistry

Immunohistochemical analyses and staining were performed according to standard procedures.[7] Used antibodies are listed in **online supplementary table 1**. After staining, tissue sections were embedded in Kaiser glycerol gelatin (Merck). Pulmonary vascular remodeling was studied by quantification of intraacinar pulmonary vessels containing a-smooth muscle actin (α-SMA)-positive cells in their walls. Vessels between and 15 and 50um external diameter and located in normal lung tissue were assessed. To assess the presence of self-reactive antigens in serum of CD19-hBTK and control mice, serum was stored and frozen until further use. Serum was subjected to cryo-sections of lungs of $RAG^{-/-}$ mice (lacking mature B and T lymphocytes). Detection Antibodies against IgG and IgM are listed in **online supplementary table 1.** Micrographs were made using a DM LB light microscope (Leica), a DFC500 camera (Leica), and Imaging for Windows Version 1.0 software (Kodak).

The Fulton index

Hearts were excised and dissected to determine the ratio of right ventricular to left ventricular and septal weight [RV/(LV + S)].

Hydroxy proline assay

Whole left lung homogenates were analyzed by quantitative hydroxyproline assay. The left lung homogenate was hydrolyzed in 6M HCl at 95°C for 20 hours. Hydroxyproline was oxidized with chloramine t, and visualized with Erlich's reagent (4-DMAB, isopropanol and perchloric acid) measured at a microplate reader at 560nm.

HEp-2

To assess the presence of self-reactive antigens in serum of CD19-hBTK and control mice, serum was stored and frozen until further use. Serum samples (1/50 diluted) were incubated for 1 hour on Kallestad human epithelial cell (HEp-2) slides (Bio-Rad Laboratories). As detection antibodies Ig F(ab')2 fragments were applied to the HEp2 slides (**online supplementary table 1**). The fluorescence

intensity of HEp2 slides was evaluated using a LSM 311 META confocal fluorescence microscope (Zeiss) and LSM Image Browser Version 4.2.0.12 software (Zeiss)

α-SMA area to total artery area

Percentage α-SMA area to total artery area was evaluated using NanoZoomer 2.0-HT slide scanner (Hamamatsu) and NDP.view 2.7.25 (Hamamatsu). Photos were analysed using Adobe Photoshop 2021 in an automated and thus independent manner.

Total fibrosis score

A pathologist (blinded for treatment) scored the Ashcroft scale (grade 1-8)[8] and the percentage of lung involvement (grade 1-5; 1 =0-10% to 6 = 75-100% of total lung involvement). The Total Fibrosis score (TFS) is the product of Ashcroft scale and lung involvement and was previously described. [9]

1. Heukels P, van Hulst JAC, van Nimwegen M, Boorsma CE, Melgert BN, van den Toorn LM*, et al.* Fibrocytes are increased in lung and peripheral blood of patients with idiopathic pulmonary fibrosis. *Respir Res* 2018, **19**(1)**:** 90.

2. Corneth OBJ, Verstappen GMP, Paulissen SMJ, de Bruijn MJW, Rip J, Lukkes M*, et al.* Enhanced Bruton's Tyrosine Kinase Activity in Peripheral Blood B Lymphocytes From Patients With Autoimmune Disease. *Arthritis Rheumatol* 2017, **69**(6)**:** 1313-1324.

3. Maas A, Dingjan GM, Grosveld F, Hendriks RW. Early arrest in B cell development in transgenic mice that express the E41K Bruton's tyrosine kinase mutant under the control of the CD19 promoter region. *J Immunol* 1999, **162**(11)**:** 6526-6533.

4. Middendorp S, Dingjan GM, Maas A, Dahlenborg K, Hendriks RW. Function of Bruton's tyrosine kinase during B cell development is partially independent of its catalytic activity. *J Immunol* 2003, **171**(11)**:** 5988-5996.

5. Kim SN, Lee J, Yang HS, Cho JW, Kwon S, Kim YB*, et al.* Dose-response Effects of Bleomycin on Inflammation and Pulmonary Fibrosis in Mice. *Toxicol Res* 2010, **26**(3)**:** 217-222.

6. McGovern TK, Robichaud A, Fereydoonzad L, Schuessler TF, Martin JG. Evaluation of respiratory system mechanics in mice using the forced oscillation technique. *J Vis Exp* 2013(75)**:** e50172.

7. GeurtsvanKessel CH, Willart MA, Bergen IM, van Rijt LS, Muskens F, Elewaut D*, et al.* Dendritic cells are crucial for maintenance of tertiary lymphoid structures in the lung of influenza virus-infected mice. *J Exp Med* 2009, **206**(11)**:** 2339-2349.

8. Ashcroft T, Simpson JM, Timbrell V. Simple method of estimating severity of pulmonary fibrosis on a numerical scale. *J Clin Pathol* 1988, **41**(4)**:** 467-470.

9. Borensztajn K, Bresser P, van der Loos C, Bot I, van den Blink B, den Bakker MA*, et al.* Protease-activated receptor-2 induces myofibroblast differentiation and tissue factor up-regulation during bleomycin-induced lung injury: potential role in pulmonary fibrosis. *Am J Pathol* 2010, **177**(6)**:** 2753-2764.

online Supplementary Table 1 Overview of extra- and intracellular antibodies use for human and mouse experiments.

marker	conjugate	company	Cat#	intra/extracellular	dilution
**HUMAN**					
**antibodies used for flow cytometry**					
IgG	FITC	BD	555786	extracellular	1:20
Btk	PE	BD	611117	intracellular	1:5
IgM	Bio	BD	555781	extracellular	1:20
CD19	PerCP-Cy5.5	BD	332780	extracellular	1:400
CD38	APC	BD	560980	extracellular	1:10
IgD	APC-H7	BD	561305	extracellular	1:10
CD27	BV421	BD	562513	extracellular	1:80
CD24	BV711	BD	563401	extracellular	1:40
CD3	AF700	eBioscience	56-0038-42	extracellular	1:40
CXCR5	PerCP5.5	BD	562781	extracellular	1:20
CD3	APC ef780	eBioscience	47-0038-42	extracellular	1:100
CD4	AF700	eBioscience	E08948-1631	extracellular	1:100
CD45RA	BV650	BD	563963	extracellular	1:40
PD1	BV786	BD	563789	extracellular	1:20
FoxP3	PE	eBioscience	12-4777-42	intracellular	1:20
CXCR3	BV711	BD	563156	extracellular	1:20
CCR4	FITC	BD	FAB1567F	extracellular	1:20
CCR6	APC	BD	560619	extracellular	1:5
CXCR5	PerCP5.5	BD	**562781**	extracellular	1:20
**HEp-2 antibodies**					
Anti-Mouse F(ab') IgG	Cy3	Jackson IR	115-166-003		
**MOUSE**					
**antibodies used for flow cytometry**					
GL7	FITC	BD	553666	extracellular	1:2000
CD95	PE-TxR	BD	562499	extracellular	1:400
IgM	Pe-Cy7	eBioscience	25-5790-82	extracellular/ intracellular	1:500
IgD	APC	eBioscience	17-5993-82	extracellular/ intracellular	1:1280
CD19	Af700	eBioscience	56-0193-82	extracellular	1:50
CD138	BV605	BD	563147	extracellular	1:400
CD3	PE-CF594	BD	562286	extracellular	1:100
CD4	Af700	eBioscience	56-0041-82	extracellular	1:200
MHC II	FITC	BD	553565	extracellular	1:200
PD-1	PE	BD	551892	extracellular	1:100
CD3	PE-CF594	BD	562286	extracellular	1:100
CD40L (CD154)	PerCP-eFl710	eBioscience	46-1541-82	extracellular	1:100
CXCR5	biotin	BD	551960	extracellular	1:50
ICOS	APC	eBioscience	17-9949-82	extracellular	1:1600

CD4	AF700	eBioscience	56-0041-82	extracellular	1:400
CD11c	APC-eFl750	eBioscience	47-0114-82	extracellular	1:200
CD11b	eFl450	eBioscience	48-0112-82	extracellular	1:200
PD-L1	BV711	BD	563369	extracellular	1:100
**HEp-2 antibodies**					
Anti-Human F(ab') IgG	Cy3	Jackson IR	109-166-003		
**Immunohisto-chemistry Lung**					
Anti-hαSMA	PE	R&D	IC1420P		
Anti-PE	AP	Rockland	600-105-387		
Anti-B220	Unlabeled	Bioceros			
Anti-Rat	AP	Sigma	A8438 – 1ML		
Anti-hCD3	Unlabeled	DAKO	A0452		
Anti-Rabbit	Biotin	Biogenex	HK326-UR		
Anti-Goat	AP	Sigma	A4187 – 1ML		
Anti-IgG	Biotin	S. Biotech	1030-08		
Anti-IgM	Biotin	S. Biotech	1020-08		
Streptavidin	AP	Biogenex	HK321-UK		
Stretavidin	PO	Biogenex	HK320-UK		

Online Supplementary Figure 1. Similar fibrosis indices in WT and CD19-hBTK mice upon bleomycin exposure.

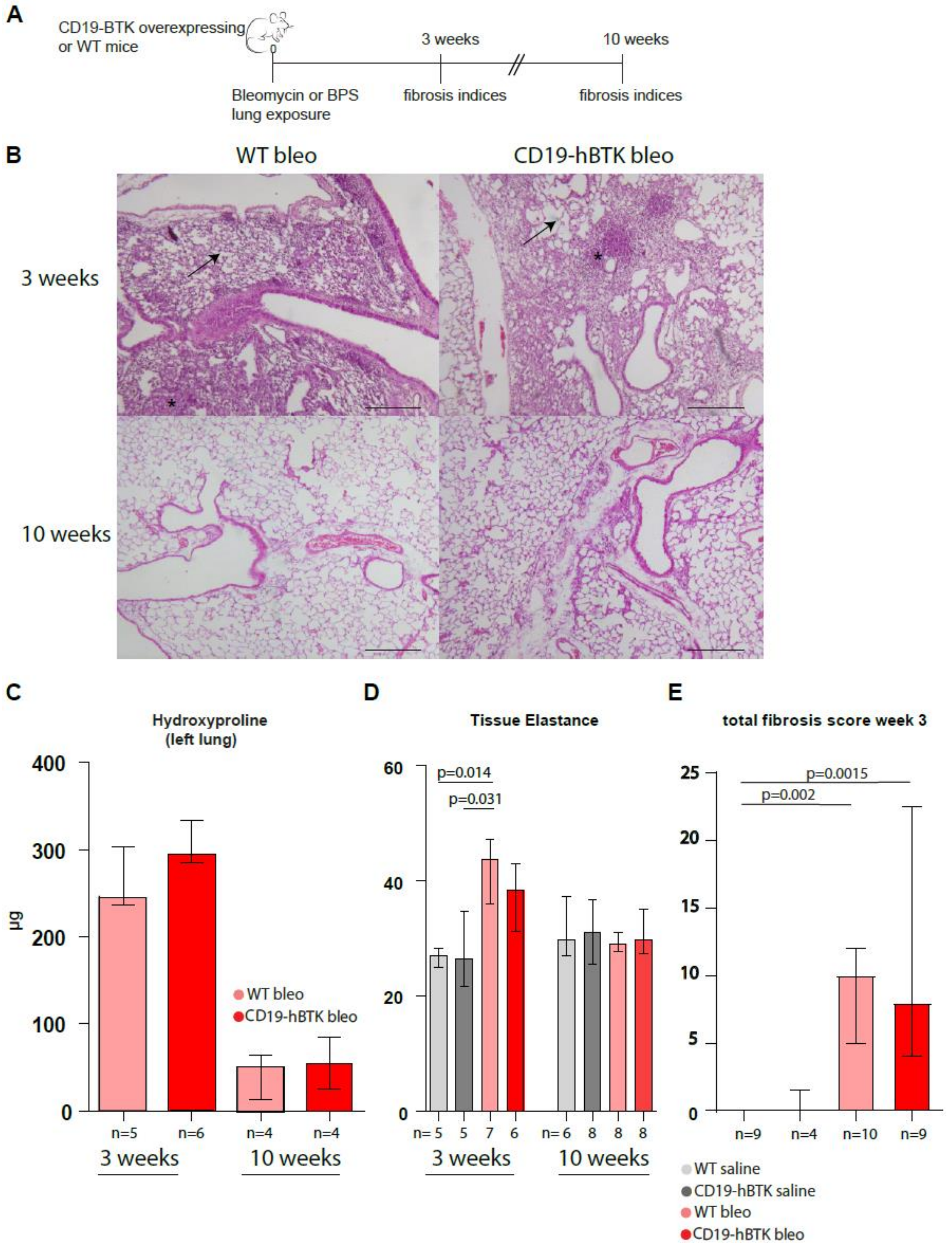

(A) Mice were sacrificed 3 and 10 weeks after saline or bleomycin exposure and analyzed for fibrosis indices. (B) Representative hematoxylin/eosin (H&E) staining of cryo-sections of lung tissue of a WT or CD19-hBTK mouse 3 and 10 weeks after bleomycin exposure. Inflammatory exudate and obvious

damage to lung architecture (asterisk) and diffuse fibrous thickening of alveolar septa (arrow). Resolution of fibrosis at 10 weeks. Magnification 40x. (C) Hydroxyproline content (µg per left lung) in WT or CD19-hBTK mice 3 and 10 weeks post bleomycin exposure (D) Tissue elastance assessed with a flexiVent FX system in WT or CD19-hBTK mice 21 days and 70 days post saline or bleomycin exposure. (E) Total fibrosis score. A pathologist (blinded for treatment) scored the Ashcroft scale (grade 1-8) and the percentage of lung involvement (grade 1-5; 1 =0-10% to 6 = 75-100% of total lung involvement). The total fibrosis score is the product of Ashcroft scale and lung involvement. The results (C-E) are shown as median (IQR), p exact values were obtained following a Kruskal-Wallis test. Number of mice used for each experiment are depicted below the graph.

Online Supplementary Figure 2. α-SMA in WT or CD19-hBTK mice 10 weeks post bleomycin exposure.

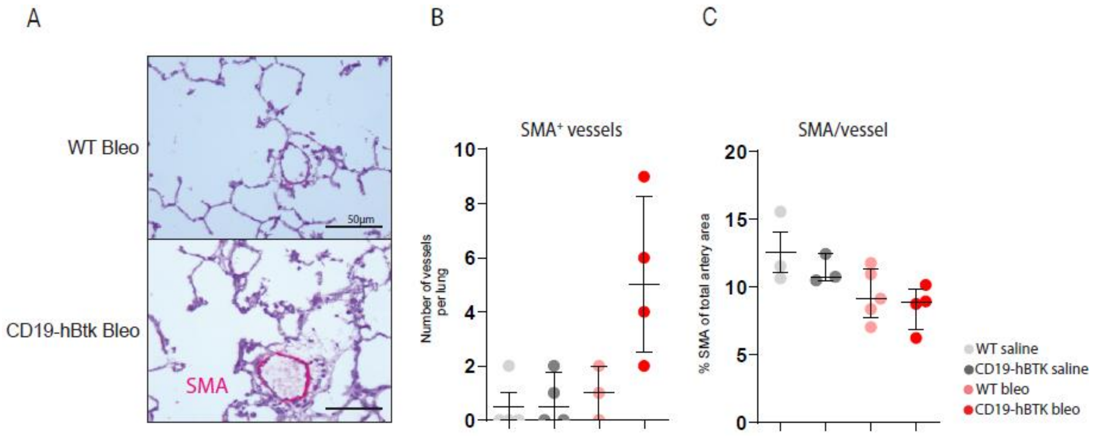

(A) Representative hematoxylin/eosin and α-smooth muscle actin (α-SMA) staining of cryo-sections of the indicated lung tissue. (B) Number of SMA-positive vessels per lung. Vessels between 15 and 50µm external diameter and located in normal lung tissue were assessed. (C) Percentage α-SMA area to total artery area of the assessed vessels. The results are shown as median (IQR), p exact values were obtained following Kruskal-Wallis test. Dots represent individual mice.

Online supplementary figure 3

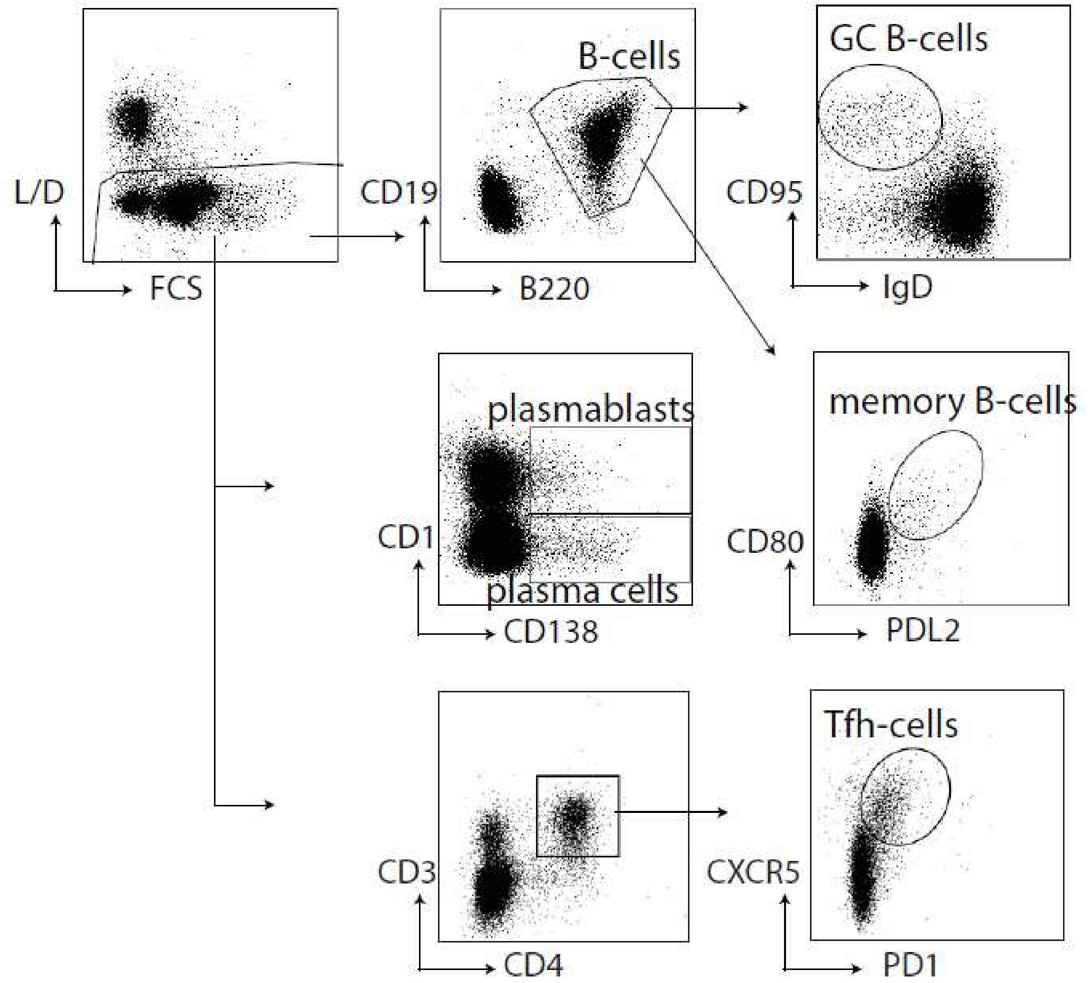

Representative gating strategy for identification of B-cell subsets and follicular T helper (Tfh)-cells in mediastinal lymph nodes (MedLN) in mice, starting with live single cells. L/D, life-death marker; FCS, forward scatter. From the total $CD19^{+}B220^{+}$ B-cell population, memory B-cells ($CD19^{+}B220^{+}CD80^{+}PDL2^{+}$), germinal center (GC) B-cells ($CD19^{+}B220^{+}IgD^{-}CD95^{+}$), plasmablasts ($CD19^{+}CD138^{+}$) and plasma cells ($CD19^{low}CD138^{+}$) were identified. From the $CD3^{+}CD4^{+}$ population, total numbers of Tfh-cells ($CD3^{+}CD4^{+}CXCR5^{+}PD1^{+}$) were identified.

Online Supplementary Figure 4: B-cell subsets and BTK expression in ILD-PH

A

	gender (male)	age (years)	mPAP (mmHg)^A	Immunomodulating therapies ^{B}	Anti fibrotics^C
HC (N=16)	10 (63%)	59 (54-64.5)	-	-	-
ILD-PH (N=17)^D	5 (29%)	63 (57.5-76)	35 (27-40)	5 (29%)	7 (41%)

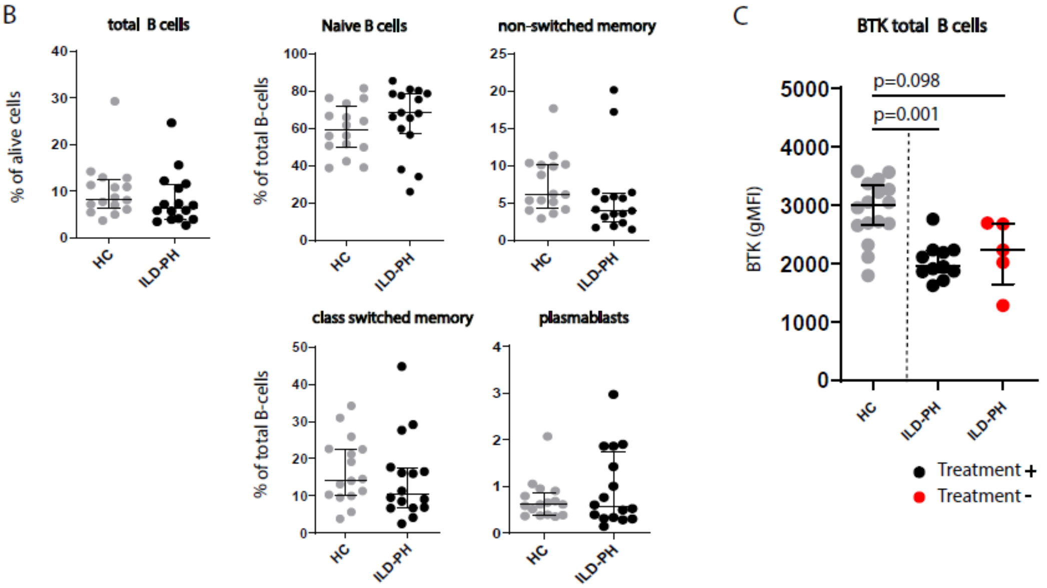

(A) Table showing the characteristics of patients with interstitial lung disease-related PH (ILD-PH) and healthy subject (HC). mPAP; mean pulmonary artery pressure. ^{A} In 6 patients only echocardiographic measurements available, showing estimated RVSP> 35mmHg and signs of RV dysfunction and no signs of left heart disease or left sided heart failure. ^{B} Four patients used prednisone >10mg/day and one patient was on azathioprine. ^{C} Nintedanib (n=3) or pirfenidone (n=4). ^{D} Ten patients with IPF, 3 patients with non-specific interstitial pneumonia, 1 patient with respiratory bronchiolitis interstitial lung disease, 1 patient with extrinsic allergic alveolitis, 1 patient with combined pulmonary fibrosis and emphysema, and 1 patient with interstitial pneumonia with autoimmune features. Continuous variables are presented as median and IQR in parentheses and categorical variable as count and percentages in parentheses.

(B) Proportions of circulating total B-cells and B-cell subpopulations (naïve B-cells (CD19$^+$IgD$^+$CD27$^-$), non-switched memory B-cells (CD19$^+$IgD$^+$CD27$^+$), and class switched memory B-cells (CD19$^+$CD27$^+$IgD$^-$IgM$^-$), and plasmablasts (CD19$^+$CD38^{hi}CD27$^+$)) in HC and patients with ILD-PH. (C) Quantification of BTK protein expression levels, shown as gMFI values of intracellular flow cytometry analysis of total B cells

in HCs and ILD-PH patients receiving immunomodulatory or anti-fibrotic treatment (*black dots*) or no treatment (*red dots*). p exact values were obtained by a Mann-Whitney U test or Kruskal-Wallis test (>2 groups). Dots represent individual values in patients.

Online Supplementary Figure 5: No correlation of cTfh cells and BTK protein in total B-cells

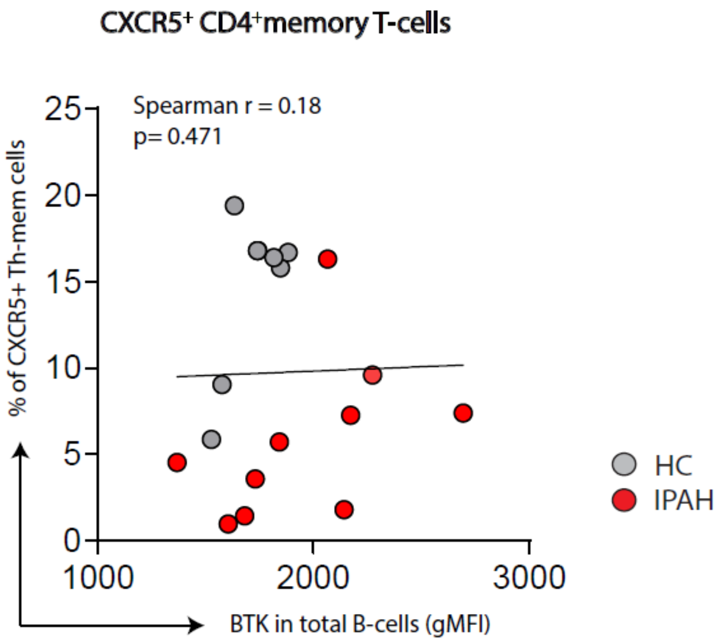

No correlation of circulating follicular T helper (cTfh) cells ($CD4^{+}CD45RA^{-}FoxP3^{low}CXCR5^{+}$) and BTK protein in total B cells in HC and patients with IPAH. Correlation coefficient was calculated using Spearman's rank method.
